# Tetra­kis[μ_2_-1,1,1,3,3,3-hexa­fluoro-2-(tri­fluoro­meth­yl)propan-2-olato]tetra­kis­(μ_3_-2-methyl­propan-2-olato)octa­copper(I)

**DOI:** 10.1107/S2056989021005429

**Published:** 2021-05-28

**Authors:** Andrew P. Purdy, Ray J. Butcher

**Affiliations:** a Chemistry Division, Code 6100, Naval Research Laboratory, 4555 Overlook Av, SW, Washington DC 20375-5342, USA; b Department of Chemistry, Howard University, 525 College Street NW, Washington DC 20059, USA

**Keywords:** crystal structure,crystal structure, Cu^I^ alkoxide, Cu_4_O_4_ square plane.

## Abstract

The title compound, [Cu_8_(C_4_H_9_O)_4_(C_4_F_9_O)_4_], crystallizes in the monoclinic space group, *P*2_1_/*n* and contains a self-assembly of two C_16_H_18_Cu_4_F_18_O_4_ units linked by bridging *tert*-butyl groups [Cu—O bonds of length 2.3779 (15) and 2.4248 (15) Å], generating a centrosymmetric dimer. The asymmetrical unit, C_16_H_18_Cu_4_F_18_O_4_, contains an almost square-planar arrangement of the four Cu atoms linked by bridging *tert*-butyl and perfluorinated *tert*-butyl groups with Cu—Cu distances ranging from 2.7108 (4) to 2.7612 (4) Å and Cu —Cu—Cu angle values close to 90° [ranging from 89.459 (10)° to 90.025 (11)°]. These dimers are further linked by weak C—H⋯F and F⋯F inter­actions. As is commonly encountered in perfluorinated *tert*-butyl groups, one of the CF_3_ groups is disordered and was refined with two equivalent conformations with occupancies of 0.74 (3) and 0.26 (3).

## Abstract 2

The structure of the title ocyacopper cluster contains two C_16_H_18_Cu_4_F_18_O_4_ units linked through a center of inversion by weak Cu—O bonds.

## Chemical context   

The structural chemistry of perfluoro­alkoxides has been the subject of much recent inter­est because of the increased acidity caused by perfluorination. Metal complexes of such species often show enhanced volatility, which makes them useful precursors to ceramic materials (Bradley, 1989 ▸) and other applications. Because of their inter­esting properties, metal complexes of these ligands have been studied extensively. Focusing on copper complexes, with suitable variants of these ligands complexes have been used to demonstrate that optically active complexes can be obtained (Cripps & Willis, 1975*a* ▸,*b* ▸). Further studies involving both perfluorinated alkoxides and copper have demonstrated their ability to obtain heterometallic complexes containing both Cu and Ba (Purdy & George 1991 ▸; Borup *et al.*, 1997 ▸) and in the use of such compounds in the oxycupration of tetra­fluoro­ethyl­ene (Ohashi *et al.*, 2017 ▸). Of particular inter­est are the alkoxide complexes of copper(I), which often form cluster compounds (Purdy & George 1995 ▸; Borup *et al.*, 1997 ▸; Purdy & George 1998 ▸; Anson *et al.*, 2005 ▸; Lieberman *et al.*, 2015 ▸). Within this set of compounds, there are those that form tetra-Cu^I^ squares bridged along the edges by oxygen donors (Greiser & Weiss, 1976 ▸; McGeary *et al.*, 1992 ▸; Terry *et al.*, 1996 ▸; Lopes *et al.*, 1997 ▸; Nikitinsky *et al.*, 2000 ▸; Håkansson *et al.*, 2000 ▸; Krossing, 2012 ▸; Bellow *et al.*, 2015 ▸). In view of the inter­esting chemistry exhibited by these alkoxide complexes containing Cu^I^, the synthesis of a mixed alkoxide complex was attempted and resulting structure of the compound is reported.
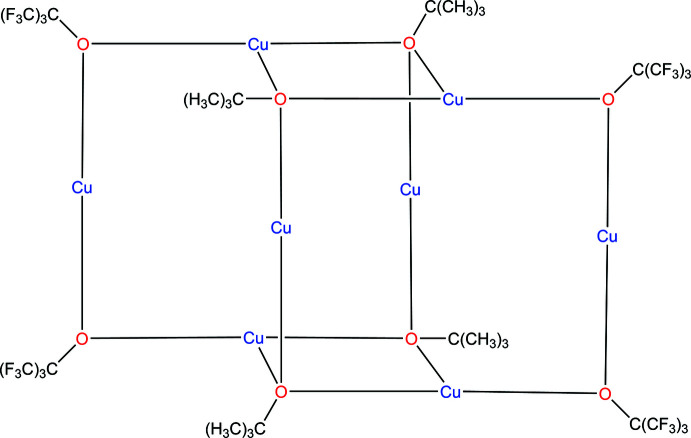


## Structural commentary   

**Figure 1:**
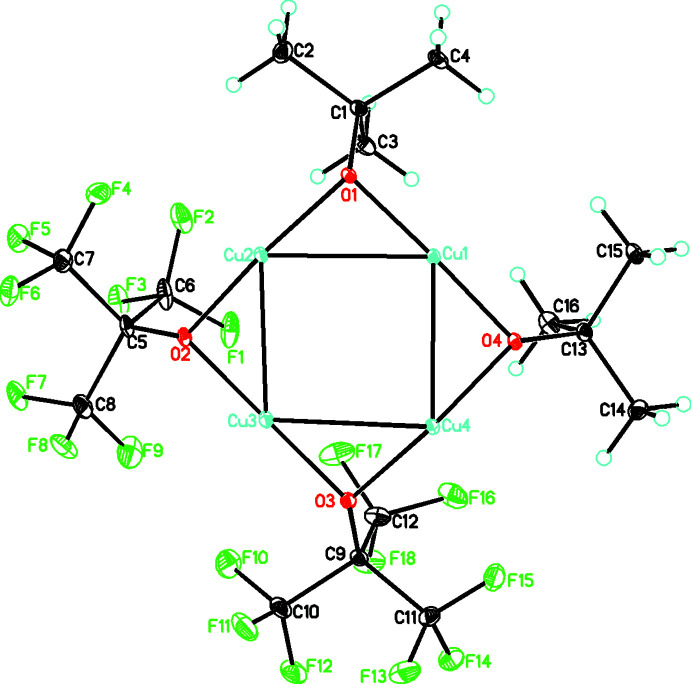
Mol­ecular diagram for the major component of **1** showing the atom labeling. Atomic displacement parameters are at the 30% level.

**Figure 2:**
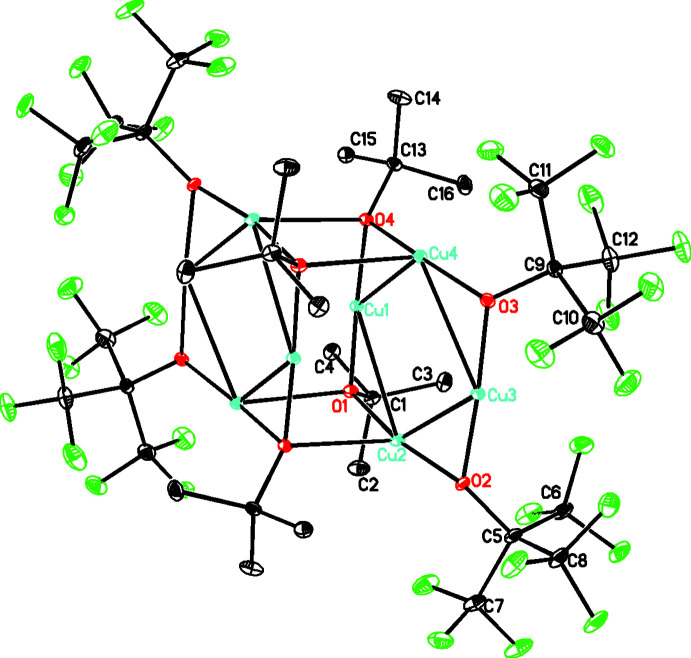
Diagram for **1** showing the association of two units into centrosymmetric dimers by weak Cu—O bonds. Hydrogen atoms omitted for clarity. Atomic displacement parameters are at the 30% level.

The title compound, C_32_H_36_Cu_8_F_36_O_8_, **1**, crystallizes in the monoclinic space group, *P*2_1_/*n*, and contains two C_16_H_18_Cu_4_F_18_O_4_ units linked through a center of inversion by weaker Cu—O bonds of length 2.3779 (15) and 2.4248 (15) Å (see Figs. 1 ▸ and 2 ▸). The central building unit, C_16_H_18_Cu_4_F_18_O_4_, (Fig. 1 ▸) contains an almost square-planar Cu_4_ metallic core linked by bridging *tert*-butyl and perfluorinated *tert*-butyl groups with Cu—Cu distances ranging from 2.7108 (4) to 2.7612 (4) Å and Cu—Cu—Cu angles ranging from 89.459 (10) to 90.025 (11)° (see Table 1 ▸). The two types of ligand are arranged around the square so that each is adjacent (*cis*) rather than opposed (*trans*) with Cu—O distances ranging from 1.8758 (16) to 1.9168 (15) Å. The coordination environment of all the Cu atoms in the asymmetric unit are different. Two of them have a two-coordinate linear geometry (Cu1 and Cu3), while two have a three-coordinate T-shaped geometry (Cu2 and Cu4). These metrical parameters are in the range found for other Cu^I^ structures with this type of core. The four oxygen donors form a plane [r.m.s. deviation of only 0.0158 (7) Å] and both Cu1 and Cu3 are in this plane while Cu2 and Cu4 deviate from this plane by 0.153 (1) and 0.129 (1) Å, respectively. Both the *t*-butyl and perfluorinated *t*-butyl groups deviate from this plane, as shown by the Cu—O—C angles which range from 118.57 (12) to 120.57 (12)° for the *t*-butyl groups and 125.64 (14) to 127.62 (14)° for the perfluorinated *t*-butyl groups, with this larger value reflecting the increased steric bulk of the latter. For both the *t*-butyl and perfluorinated *t*-butyl groups, this deviation is on the same side of the Cu_4_O_4_ plane to allow for the association of the two C_16_H_18_Cu_4_F_18_O_4_ units into the dimer mentioned above (see Fig. 2 ▸).

## Supra­molecular features   

**Figure 3:**
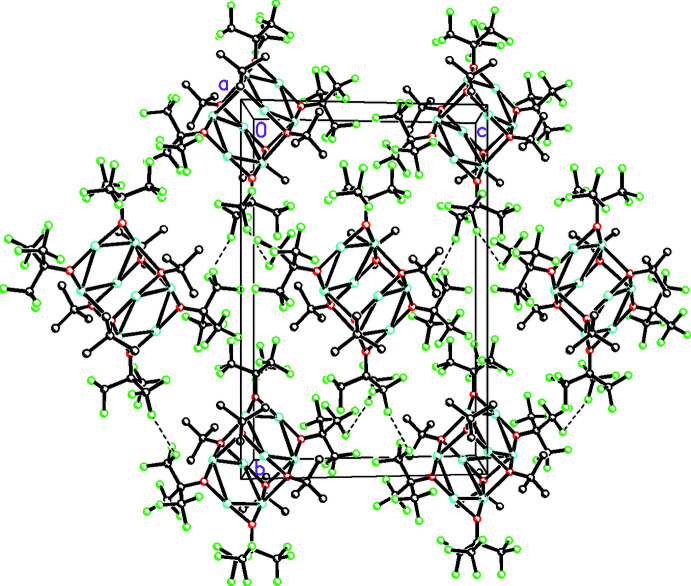
Packing diagram viewed along the *a* axis.

In addition to the weak Cu—O inter­actions associating the C_16_H_18_Cu_4_F_18_O_4_ units into dimers, there are also intra­dimer C—H⋯F inter­actions (see Table 2 ▸ and Fig. 2 ▸). These dimers are further linked by weak inter­dimer C—H⋯F and F⋯F inter­actions. While intra­dimer F⋯F are numerous, there are very few inter­dimer C—H⋯F or F⋯F inter­actions, which reflects the fact that this compound was originally isolated by sublimation from the reaction mixture. The overall packing is shown in Fig. 3 ▸.

## Database survey   

In the literature there are six examples of structures containing a square-planar Cu_4_O_4_ arrangement and they divide into two groups. In the first group, this Cu_4_O_4_ arrangement is isolated owing to the steric bulk of the O substituents [JUVKUG (McGeary *et al.*, 1992 ▸); ZUTCIA (Terry *et al.*, 1996 ▸); QEMCUG (Nikitinsky *et al.*, 2000 ▸); GEQCUC (Krossing, 2012 ▸),] while in the second group these units associate into dimers [CUTBUX (Greiser & Weiss, 1976 ▸); CUTBUX01 (Håkansson *et al.*, 2000 ▸)]. Inter­estingly, in these two groups, one contains a structure where all the substituents are *t*-butyl groups [two polymorphs of the *tert*-butyl derivative (Greiser & Weiss, 1976 ▸; Håkansson *et al.*, 2000 ▸)] and thus the C_16_H_36_Cu_4_O_4_ units associate into dimers, while the other group contains a structure where all the substituents are perfluorinated *t*-butyl groups and this has an isolated C_16_F_36_Cu_4_O_4_ unit. Thus **1**, which has two of each type in a *cis* arrangement, has just enough steric freedom to associate into these dimeric units.

The dimerization of **1** and those for both CUTBUX and CUTBUX01 have the same arrangement where they associate *via* a crystallographic center of inversion (see Fig. 2 ▸).

## Synthesis and crystallization   

Copper(I) *t*-butoxide (0.25 g) was mixed with perfluoro-*t*-butanol (1.02 g) in a small amount of dry heptane under an inert atmosphere, and stirred for 4 d. The mixture was pumped to dryness and sublimed under vacuum at 333–373 K. A portion was sealed into an NMR tube with C_6_D_6_, and the spectrum shows both normal and fluorinated *t*-butyl groups. After many years, the NMR tube was opened and crystals were isolated.

## Refinement   

Crystal data, data collection and structure refinement details are summarized in Table 3 ▸. One of the CF_3_ groups was found to be disordered and was refined with two equivalent conformations with occupancies of 0.74 (3) and 0.26 (3). The H atoms were refined in idealized positions using a riding model with atomic displacement parameters of *U*_iso_(H) = 1.5*U*_eq_(C) for CH_3_, with C—H distances of 0.98 Å.

## Supplementary Material

Crystal structure: contains datablock(s) I. DOI: 10.1107/S2056989021005429/zn2004sup1.cif

Structure factors: contains datablock(s) I. DOI: 10.1107/S2056989021005429/zn2004Isup2.hkl

CCDC reference: 2085512

Additional supporting information:  crystallographic information; 3D view; checkCIF report

## Figures and Tables

**Table 1:** Selected geometric parameters (Å, °)

Cu1—O1	1.8488 (15)	Cu2—O4^i^	2.3779 (15)
Cu1—O4	1.8496 (15)	Cu2—Cu3	2.7172 (4)
Cu1—Cu4	2.7108 (4)	Cu3—O3	1.8758 (16)
Cu1—Cu2	2.7526 (3)	Cu3—O2	1.8770 (15)
Cu1—Cu1^i^	2.9452 (5)	Cu3—Cu4	2.7612 (4)
Cu1—Cu4^i^	2.9816 (4)	Cu4—O4	1.9001 (14)
Cu2—O1	1.8956 (14)	Cu4—O3	1.9137 (15)
Cu2—O2	1.9168 (15)	Cu4—O1^i^	2.4248 (15)
			
O1—Cu1—O4	177.42 (7)	O3—Cu3—O2	176.74 (7)
O1—Cu2—O2	170.02 (7)	O4—Cu4—O3	171.27 (7)
O1—Cu2—O4^i^	85.40 (6)	O4—Cu4—O1^i^	83.99 (6)
O2—Cu2—O4^i^	103.39 (6)	O3—Cu4—O1^i^	103.85 (6)

Symmetry code: (i) -x+1, -y+1, -z+1.

**Table 2:** Hydrogen-bond geometry (Å, °)

*D*—H⋯*A*	*D*—H	H⋯*A*	*D*⋯*A*	*D*—H⋯*A*
C3—H3*A*⋯F2	0.98	2.51	3.457 (7)	164
C3—H3*A*⋯F2*A*	0.98	2.56	3.539 (18)	173
C14—H14*A*⋯F15	0.98	2.63	3.571 (3)	162
C14—H14*B*⋯F4^i^	0.98	2.58	3.554 (4)	173
C15—H15*A*⋯O2^i^	0.98	2.58	3.438 (3)	146
C15—H15*C*⋯F3*A* ^ii^	0.98	2.64	3.581 (18)	161
C16—H16*A*⋯F16	0.98	2.57	3.536 (3)	170

Symmetry codes: (i) -x+1, -y+1, -z+1; (ii) -x+{\script{1\over 2}}, y+{\script{1\over 2}}, -z+{\script{1\over 2}}.

**Table 3:** Experimental details

Crystal data	
Chemical formula	[Cu_8_(C_4_H_9_O)_4_(C_4_F_9_O)_4_]
*M*_r_	1740.93
Crystal system, space group	Monoclinic, *P*2_1_/*n*
Temperature (K)	100
*a*, *b*, *c* (Å)	10.3302 (2), 19.5926 (5), 13.1516 (3)
β (°)	101.004 (1)
*V* (Å^3^)	2612.88 (10)
*Z*	2
Radiation type	Mo *K*α
μ (mm^−1^)	3.36
Crystal size (mm)	0.40 × 0.24 × 0.16

Data collection	
Diffractometer	Bruker APEXII CCD
Absorption correction	Multi-scan (*SADABS*; Bruker, 2016 ▸)
*T*_min_, *T*_max_	0.581, 0.747
No. of measured, independent and observed [*I* > 2σ(*I*)] reflections	39864, 12618, 9717
*R*_int_	0.032
(sin θ/λ)_max_ (Å^−1^)	0.834

Refinement	
*R*[*F* ^2^ > 2σ(*F* ^2^)], *wR*(*F* ^2^), *S*	0.040, 0.103, 1.02
No. of reflections	12618
No. of parameters	423
No. of restraints	138
H-atom treatment	H-atom parameters constrained
Δρ_max_, Δρ_min_ (e Å^−3^)	1.25, −1.09

Computer programs: *APEX2* and *SAINT* (Bruker, 2016 ▸), *SHELXT* (Sheldrick 2015*a* ▸), *SHELXL2018/3* (Sheldrick, 2015*b* ▸), and *SHELXTL* (Sheldrick 2008 ▸).

## supplementary crystallographic information

### Crystal data

**Table:**

[Cu_8_(C_4_H_9_O)_4_(C_4_F_9_O)_4_]	*F*(000) = 1696
*M**_r_* = 1740.93	*D*_x_ = 2.213 Mg m^−^^3^
Monoclinic, *P*2_1_/*n*	Mo *K*α radiation, λ = 0.71073 Å
*a* = 10.3302 (2) Å	Cell parameters from 9832 reflections
*b* = 19.5926 (5) Å	θ = 2.6–36.3°
*c* = 13.1516 (3) Å	µ = 3.36 mm^−^^1^
β = 101.004 (1)°	*T* = 100 K
*V* = 2612.88 (10) Å^3^	Thick needle, colorless
*Z* = 2	0.40 × 0.24 × 0.16 mm

### Data collection

**Table:**

Bruker APEXII CCD diffractometer	9717 reflections with *I* > 2σ(*I*)
φ and ω scans	*R*_int_ = 0.032
Absorption correction: multi-scan (SADABS; Bruker, 2016)	θ_max_ = 36.3°, θ_min_ = 2.5°
*T*_min_ = 0.581, *T*_max_ = 0.747	*h* = −17→16
39864 measured reflections	*k* = −32→32
12618 independent reflections	*l* = −21→14

### Refinement

**Table:**

Refinement on *F*^2^	138 restraints
Least-squares matrix: full	Hydrogen site location: inferred from neighbouring sites
*R*[*F*^2^ > 2σ(*F*^2^)] = 0.040	H-atom parameters constrained
*wR*(*F*^2^) = 0.103	*w* = 1/[σ^2^(*F*_o_^2^) + (0.0408*P*)^2^ + 4.799*P*] where *P* = (*F*_o_^2^ + 2*F*_c_^2^)/3
*S* = 1.02	(Δ/σ)_max_ = 0.001
12618 reflections	Δρ_max_ = 1.25 e Å^−^^3^
423 parameters	Δρ_min_ = −1.09 e Å^−^^3^

### Special details

**Table:**

Geometry. All esds (except the esd in the dihedral angle between two l.s. planes) are estimated using the full covariance matrix. The cell esds are taken into account individually in the estimation of esds in distances, angles and torsion angles; correlations between esds in cell parameters are only used when they are defined by crystal symmetry. An approximate (isotropic) treatment of cell esds is used for estimating esds involving l.s. planes.

### Fractional atomic coordinates and isotropic or equivalent isotropic displacement parameters (Å^2^)

**Table:**

	*x*	*y*	*z*	*U*_iso_*/*U*_eq_	Occ. (<1)
Cu1	0.36569 (2)	0.48167 (2)	0.44719 (2)	0.01322 (5)	
Cu2	0.50985 (2)	0.37115 (2)	0.53468 (2)	0.01558 (5)	
Cu3	0.67148 (3)	0.38356 (2)	0.39516 (2)	0.01552 (5)	
Cu4	0.54489 (2)	0.50343 (2)	0.32457 (2)	0.01505 (5)	
C6	0.5539 (8)	0.2154 (3)	0.4265 (7)	0.0348 (14)	0.74 (3)
F1	0.5195 (10)	0.2498 (4)	0.3398 (6)	0.0482 (16)	0.74 (3)
F2	0.4463 (6)	0.2130 (3)	0.4711 (10)	0.0458 (16)	0.74 (3)
F3	0.5820 (9)	0.1514 (3)	0.4055 (8)	0.0457 (17)	0.74 (3)
C6A	0.569 (2)	0.2219 (11)	0.402 (2)	0.039 (3)	0.26 (3)
F1A	0.548 (2)	0.2606 (11)	0.3199 (19)	0.048 (3)	0.26 (3)
F2A	0.4536 (16)	0.2137 (10)	0.433 (2)	0.046 (3)	0.26 (3)
F3A	0.6044 (18)	0.1603 (9)	0.373 (2)	0.046 (3)	0.26 (3)
F4	0.5764 (2)	0.24802 (10)	0.65140 (18)	0.0449 (5)	
F5	0.6632 (2)	0.15454 (9)	0.61016 (19)	0.0458 (5)	
F6	0.78728 (19)	0.23828 (10)	0.67233 (15)	0.0408 (4)	
F7	0.84303 (18)	0.17070 (9)	0.48863 (17)	0.0418 (5)	
F8	0.89770 (17)	0.27610 (9)	0.51149 (19)	0.0429 (5)	
F9	0.7930 (3)	0.24249 (13)	0.36435 (18)	0.0603 (7)	
F10	0.8330 (3)	0.33139 (11)	0.2327 (2)	0.0556 (6)	
F11	0.93598 (18)	0.42150 (13)	0.29185 (16)	0.0502 (6)	
F12	0.9025 (2)	0.39975 (13)	0.12788 (16)	0.0472 (5)	
F13	0.8467 (2)	0.53540 (11)	0.18854 (17)	0.0468 (5)	
F14	0.7351 (2)	0.49850 (11)	0.04494 (14)	0.0411 (4)	
F15	0.6371 (2)	0.54858 (11)	0.15382 (17)	0.0465 (5)	
F16	0.50493 (19)	0.43350 (13)	0.09123 (16)	0.0505 (6)	
F17	0.5644 (2)	0.34473 (12)	0.18442 (16)	0.0525 (6)	
F18	0.6462 (2)	0.36536 (12)	0.04940 (15)	0.0488 (5)	
O1	0.35045 (14)	0.41607 (8)	0.54527 (12)	0.0150 (2)	
O2	0.65525 (14)	0.32124 (7)	0.50039 (12)	0.0155 (3)	
O3	0.68844 (15)	0.44984 (8)	0.29561 (11)	0.0165 (3)	
O4	0.38626 (14)	0.54477 (7)	0.34687 (11)	0.0139 (2)	
C1	0.22828 (19)	0.37709 (11)	0.53597 (18)	0.0178 (4)	
C2	0.2449 (3)	0.32974 (15)	0.6293 (2)	0.0303 (5)	
H2A	0.317793	0.298079	0.627315	0.045*	
H2B	0.163328	0.303840	0.627592	0.045*	
H2C	0.264175	0.356809	0.693052	0.045*	
C3	0.2035 (2)	0.33683 (13)	0.4354 (2)	0.0263 (5)	
H3A	0.278294	0.306346	0.433756	0.039*	
H3B	0.193375	0.368419	0.376656	0.039*	
H3C	0.122870	0.309737	0.430963	0.039*	
C4	0.1165 (2)	0.42778 (13)	0.5378 (2)	0.0229 (4)	
H4A	0.104906	0.456535	0.475715	0.034*	
H4B	0.138111	0.456534	0.599653	0.034*	
H4C	0.034607	0.402801	0.539074	0.034*	
C5	0.6712 (2)	0.25132 (11)	0.49893 (19)	0.0203 (4)	
C7	0.6734 (3)	0.22204 (13)	0.6093 (2)	0.0307 (5)	
C8	0.8043 (3)	0.23447 (14)	0.4662 (2)	0.0309 (5)	
C9	0.7179 (2)	0.43721 (12)	0.19933 (16)	0.0185 (4)	
C10	0.8520 (3)	0.39842 (17)	0.2129 (2)	0.0328 (6)	
C11	0.7320 (3)	0.50577 (14)	0.1441 (2)	0.0279 (5)	
C12	0.6092 (3)	0.39317 (16)	0.1314 (2)	0.0306 (5)	
C13	0.2764 (2)	0.55606 (11)	0.26043 (16)	0.0177 (3)	
C14	0.3218 (3)	0.60780 (16)	0.1893 (2)	0.0296 (5)	
H14A	0.397446	0.589458	0.163124	0.044*	
H14B	0.347723	0.650043	0.227719	0.044*	
H14C	0.249709	0.617393	0.130988	0.044*	
C15	0.1606 (2)	0.58315 (12)	0.30501 (18)	0.0216 (4)	
H15A	0.187194	0.624869	0.344617	0.032*	
H15B	0.133394	0.548669	0.350682	0.032*	
H15C	0.086657	0.593322	0.248369	0.032*	
C16	0.2385 (3)	0.48899 (14)	0.2039 (2)	0.0273 (5)	
H16A	0.315517	0.469958	0.180408	0.041*	
H16B	0.167918	0.497218	0.143911	0.041*	
H16C	0.207776	0.456652	0.250932	0.041*	

### Atomic displacement parameters (Å^2^)

**Table:**

	*U*^11^	*U*^22^	*U*^33^	*U*^12^	*U*^13^	*U*^23^
Cu1	0.01056 (9)	0.01428 (10)	0.01405 (10)	0.00057 (8)	0.00039 (7)	0.00096 (8)
Cu2	0.01264 (10)	0.01438 (10)	0.01934 (12)	0.00278 (8)	0.00208 (8)	0.00314 (8)
Cu3	0.01483 (10)	0.01579 (11)	0.01540 (11)	0.00344 (8)	0.00154 (8)	0.00048 (8)
Cu4	0.01328 (10)	0.01641 (11)	0.01549 (11)	0.00283 (8)	0.00280 (8)	0.00206 (8)
C6	0.032 (2)	0.0152 (16)	0.047 (3)	0.0041 (15)	−0.020 (2)	−0.0064 (18)
F1	0.056 (3)	0.0304 (19)	0.042 (2)	0.0023 (19)	−0.030 (2)	−0.0024 (15)
F2	0.0227 (14)	0.0280 (14)	0.079 (4)	−0.0057 (11)	−0.010 (2)	0.001 (2)
F3	0.050 (3)	0.0174 (14)	0.058 (3)	0.0031 (15)	−0.020 (2)	−0.0149 (16)
C6A	0.032 (5)	0.024 (5)	0.054 (6)	−0.001 (4)	−0.010 (5)	−0.013 (5)
F1A	0.052 (6)	0.031 (5)	0.053 (7)	−0.008 (5)	−0.013 (5)	−0.013 (4)
F2A	0.020 (4)	0.035 (4)	0.075 (7)	−0.005 (3)	−0.010 (5)	−0.011 (6)
F3A	0.041 (5)	0.024 (4)	0.067 (8)	−0.002 (4)	−0.004 (5)	−0.015 (5)
F4	0.0464 (11)	0.0339 (9)	0.0613 (13)	0.0087 (8)	0.0279 (10)	0.0189 (9)
F5	0.0423 (10)	0.0165 (7)	0.0776 (15)	0.0008 (7)	0.0091 (10)	0.0158 (8)
F6	0.0406 (10)	0.0381 (9)	0.0374 (9)	0.0014 (8)	−0.0084 (8)	0.0112 (8)
F7	0.0334 (9)	0.0229 (7)	0.0666 (13)	0.0157 (7)	0.0029 (8)	−0.0033 (8)
F8	0.0207 (7)	0.0298 (8)	0.0800 (15)	0.0017 (6)	0.0143 (8)	−0.0032 (9)
F9	0.0824 (17)	0.0595 (14)	0.0444 (12)	0.0375 (13)	0.0252 (12)	−0.0002 (10)
F10	0.0651 (15)	0.0383 (11)	0.0698 (15)	0.0214 (10)	0.0289 (12)	0.0052 (10)
F11	0.0250 (8)	0.0819 (17)	0.0421 (11)	0.0136 (9)	0.0026 (7)	0.0009 (11)
F12	0.0375 (10)	0.0691 (14)	0.0407 (10)	0.0165 (10)	0.0220 (8)	0.0005 (10)
F13	0.0472 (11)	0.0495 (12)	0.0449 (11)	−0.0181 (9)	0.0121 (9)	0.0044 (9)
F14	0.0480 (11)	0.0556 (12)	0.0228 (8)	0.0045 (9)	0.0145 (7)	0.0077 (8)
F15	0.0577 (12)	0.0428 (11)	0.0435 (11)	0.0195 (9)	0.0212 (9)	0.0166 (9)
F16	0.0284 (9)	0.0821 (17)	0.0374 (10)	−0.0024 (10)	−0.0027 (7)	−0.0098 (11)
F17	0.0647 (14)	0.0565 (13)	0.0386 (10)	−0.0348 (11)	0.0158 (10)	−0.0149 (9)
F18	0.0568 (13)	0.0630 (14)	0.0296 (9)	−0.0166 (11)	0.0154 (9)	−0.0214 (9)
O1	0.0096 (5)	0.0171 (6)	0.0180 (6)	0.0002 (5)	0.0017 (5)	0.0026 (5)
O2	0.0148 (6)	0.0107 (5)	0.0199 (7)	0.0026 (5)	0.0004 (5)	−0.0007 (5)
O3	0.0163 (6)	0.0205 (7)	0.0134 (6)	0.0037 (5)	0.0042 (5)	0.0009 (5)
O4	0.0109 (5)	0.0159 (6)	0.0139 (6)	0.0014 (5)	−0.0005 (4)	0.0007 (5)
C1	0.0110 (7)	0.0182 (8)	0.0244 (10)	−0.0015 (6)	0.0035 (6)	0.0026 (7)
C2	0.0235 (11)	0.0303 (12)	0.0357 (13)	−0.0050 (9)	0.0023 (9)	0.0128 (10)
C3	0.0172 (9)	0.0272 (11)	0.0335 (12)	−0.0022 (8)	0.0028 (8)	−0.0092 (9)
C4	0.0116 (8)	0.0265 (10)	0.0310 (11)	0.0009 (7)	0.0048 (7)	−0.0010 (9)
C5	0.0153 (8)	0.0124 (8)	0.0298 (11)	0.0022 (6)	−0.0047 (7)	−0.0009 (7)
C7	0.0304 (12)	0.0171 (10)	0.0445 (15)	0.0028 (9)	0.0068 (11)	0.0084 (10)
C8	0.0290 (12)	0.0221 (11)	0.0420 (15)	0.0116 (9)	0.0076 (10)	−0.0020 (10)
C9	0.0170 (8)	0.0235 (9)	0.0155 (8)	0.0014 (7)	0.0043 (6)	−0.0017 (7)
C10	0.0242 (11)	0.0480 (16)	0.0288 (12)	0.0115 (11)	0.0113 (9)	0.0006 (11)
C11	0.0331 (12)	0.0318 (12)	0.0209 (10)	0.0015 (10)	0.0103 (9)	0.0046 (9)
C12	0.0294 (12)	0.0389 (14)	0.0253 (11)	−0.0094 (10)	0.0098 (9)	−0.0126 (10)
C13	0.0139 (8)	0.0229 (9)	0.0144 (8)	0.0024 (7)	−0.0020 (6)	0.0016 (7)
C14	0.0236 (11)	0.0404 (14)	0.0238 (11)	0.0024 (10)	0.0017 (8)	0.0150 (10)
C15	0.0136 (8)	0.0259 (10)	0.0236 (10)	0.0047 (7)	−0.0008 (7)	0.0000 (8)
C16	0.0235 (10)	0.0328 (12)	0.0224 (10)	0.0004 (9)	−0.0033 (8)	−0.0094 (9)

### Geometric parameters (Å, º)

**Table:**

Cu1—O1	1.8488 (15)	F16—C12	1.359 (4)
Cu1—O4	1.8496 (15)	F17—C12	1.313 (4)
Cu1—Cu4	2.7108 (4)	F18—C12	1.328 (3)
Cu1—Cu2	2.7526 (3)	O1—C1	1.460 (2)
Cu1—Cu1^i^	2.9452 (5)	O2—C5	1.380 (2)
Cu1—Cu4^i^	2.9816 (4)	O3—C9	1.380 (3)
Cu2—O1	1.8956 (14)	O4—C13	1.461 (2)
Cu2—O2	1.9168 (15)	C1—C3	1.519 (3)
Cu2—O4^i^	2.3779 (15)	C1—C2	1.522 (3)
Cu2—Cu3	2.7172 (4)	C1—C4	1.527 (3)
Cu3—O3	1.8758 (16)	C2—H2A	0.9800
Cu3—O2	1.8770 (15)	C2—H2B	0.9800
Cu3—Cu4	2.7612 (4)	C2—H2C	0.9800
Cu4—O4	1.9001 (14)	C3—H3A	0.9800
Cu4—O3	1.9137 (15)	C3—H3B	0.9800
Cu4—O1^i^	2.4248 (15)	C3—H3C	0.9800
C6—F1	1.314 (6)	C4—H4A	0.9800
C6—F3	1.329 (6)	C4—H4B	0.9800
C6—F2	1.353 (7)	C4—H4C	0.9800
C6—C5	1.558 (7)	C5—C8	1.553 (4)
C6A—F1A	1.302 (16)	C5—C7	1.556 (4)
C6A—F3A	1.335 (17)	C9—C11	1.547 (3)
C6A—F2A	1.342 (16)	C9—C12	1.556 (3)
C6A—C5	1.60 (2)	C9—C10	1.560 (3)
F4—C7	1.335 (3)	C13—C14	1.513 (3)
F5—C7	1.327 (3)	C13—C16	1.524 (3)
F6—C7	1.342 (3)	C13—C15	1.525 (3)
F7—C8	1.328 (3)	C14—H14A	0.9800
F8—C8	1.316 (4)	C14—H14B	0.9800
F9—C8	1.332 (4)	C14—H14C	0.9800
F10—C10	1.360 (4)	C15—H15A	0.9800
F11—C10	1.301 (4)	C15—H15B	0.9800
F12—C10	1.321 (3)	C15—H15C	0.9800
F13—C11	1.348 (4)	C16—H16A	0.9800
F14—C11	1.319 (3)	C16—H16B	0.9800
F15—C11	1.315 (3)	C16—H16C	0.9800
			
O1—Cu1—O4	177.42 (7)	C1—C2—H2C	109.5
O1—Cu1—Cu4	133.09 (5)	H2A—C2—H2C	109.5
O4—Cu1—Cu4	44.45 (4)	H2B—C2—H2C	109.5
O1—Cu1—Cu2	43.35 (4)	C1—C3—H3A	109.5
O4—Cu1—Cu2	134.21 (5)	C1—C3—H3B	109.5
Cu4—Cu1—Cu2	89.771 (10)	H3A—C3—H3B	109.5
O1—Cu1—Cu1^i^	92.20 (5)	C1—C3—H3C	109.5
O4—Cu1—Cu1^i^	86.98 (4)	H3A—C3—H3C	109.5
Cu4—Cu1—Cu1^i^	63.468 (10)	H3B—C3—H3C	109.5
Cu2—Cu1—Cu1^i^	66.971 (10)	C1—C4—H4A	109.5
O1—Cu1—Cu4^i^	54.33 (5)	C1—C4—H4B	109.5
O4—Cu1—Cu4^i^	126.65 (5)	H4A—C4—H4B	109.5
Cu4—Cu1—Cu4^i^	117.900 (10)	C1—C4—H4C	109.5
Cu2—Cu1—Cu4^i^	67.494 (9)	H4A—C4—H4C	109.5
Cu1^i^—Cu1—Cu4^i^	54.432 (9)	H4B—C4—H4C	109.5
O1—Cu2—O2	170.02 (7)	O2—C5—C8	109.21 (19)
O1—Cu2—O4^i^	85.40 (6)	O2—C5—C7	109.5 (2)
O2—Cu2—O4^i^	103.39 (6)	C8—C5—C7	108.9 (2)
O1—Cu2—Cu3	131.48 (5)	O2—C5—C6	112.1 (3)
O2—Cu2—Cu3	43.67 (5)	C8—C5—C6	111.2 (4)
O4^i^—Cu2—Cu3	96.99 (4)	C7—C5—C6	105.9 (4)
O1—Cu2—Cu1	42.03 (5)	O2—C5—C6A	107.8 (8)
O2—Cu2—Cu1	133.53 (5)	C8—C5—C6A	100.8 (9)
O4^i^—Cu2—Cu1	82.49 (4)	C7—C5—C6A	120.0 (10)
Cu3—Cu2—Cu1	90.025 (11)	F5—C7—F4	108.0 (2)
O3—Cu3—O2	176.74 (7)	F5—C7—F6	107.0 (2)
O3—Cu3—Cu2	133.20 (5)	F4—C7—F6	107.0 (3)
O2—Cu3—Cu2	44.85 (4)	F5—C7—C5	112.9 (3)
O3—Cu3—Cu4	43.77 (5)	F4—C7—C5	111.6 (2)
O2—Cu3—Cu4	134.27 (5)	F6—C7—C5	110.1 (2)
Cu2—Cu3—Cu4	89.459 (10)	F8—C8—F7	108.6 (2)
O4—Cu4—O3	171.27 (7)	F8—C8—F9	107.4 (3)
O4—Cu4—O1^i^	83.99 (6)	F7—C8—F9	107.4 (2)
O3—Cu4—O1^i^	103.85 (6)	F8—C8—C5	110.7 (2)
O4—Cu4—Cu1	42.97 (4)	F7—C8—C5	112.6 (2)
O3—Cu4—Cu1	132.47 (5)	F9—C8—C5	109.9 (2)
O1^i^—Cu4—Cu1	86.87 (4)	O3—C9—C11	109.37 (19)
O4—Cu4—Cu3	132.66 (5)	O3—C9—C12	111.40 (18)
O3—Cu4—Cu3	42.69 (5)	C11—C9—C12	109.5 (2)
O1^i^—Cu4—Cu3	101.37 (4)	O3—C9—C10	109.20 (18)
Cu1—Cu4—Cu3	89.976 (11)	C11—C9—C10	108.4 (2)
O4—Cu4—Cu1^i^	85.04 (4)	C12—C9—C10	108.9 (2)
O3—Cu4—Cu1^i^	98.69 (5)	F11—C10—F12	111.0 (3)
O1^i^—Cu4—Cu1^i^	38.27 (4)	F11—C10—F10	106.3 (3)
Cu1—Cu4—Cu1^i^	62.100 (10)	F12—C10—F10	106.0 (3)
Cu3—Cu4—Cu1^i^	73.077 (9)	F11—C10—C9	111.4 (2)
F1—C6—F3	109.7 (5)	F12—C10—C9	112.3 (2)
F1—C6—F2	106.6 (5)	F10—C10—C9	109.6 (2)
F3—C6—F2	106.7 (5)	F15—C11—F14	108.9 (2)
F1—C6—C5	110.4 (5)	F15—C11—F13	107.0 (3)
F3—C6—C5	112.2 (5)	F14—C11—F13	106.8 (2)
F2—C6—C5	111.0 (5)	F15—C11—C9	111.7 (2)
F1A—C6A—F3A	107.8 (15)	F14—C11—C9	113.1 (2)
F1A—C6A—F2A	107.6 (15)	F13—C11—C9	108.9 (2)
F3A—C6A—F2A	106.6 (15)	F17—C12—F18	108.6 (3)
F1A—C6A—C5	115.6 (16)	F17—C12—F16	107.0 (2)
F3A—C6A—C5	112.1 (15)	F18—C12—F16	104.6 (2)
F2A—C6A—C5	106.7 (15)	F17—C12—C9	112.7 (2)
C1—O1—Cu1	119.50 (12)	F18—C12—C9	114.0 (2)
C1—O1—Cu2	120.00 (13)	F16—C12—C9	109.4 (2)
Cu1—O1—Cu2	94.63 (7)	O4—C13—C14	107.23 (17)
C1—O1—Cu4^i^	131.39 (12)	O4—C13—C16	109.77 (18)
Cu1—O1—Cu4^i^	87.39 (6)	C14—C13—C16	110.9 (2)
Cu2—O1—Cu4^i^	94.44 (6)	O4—C13—C15	107.65 (17)
C5—O2—Cu3	127.43 (15)	C14—C13—C15	111.3 (2)
C5—O2—Cu2	127.62 (14)	C16—C13—C15	109.89 (19)
Cu3—O2—Cu2	91.48 (6)	C13—C14—H14A	109.5
C9—O3—Cu3	125.64 (14)	C13—C14—H14B	109.5
C9—O3—Cu4	126.58 (13)	H14A—C14—H14B	109.5
Cu3—O3—Cu4	93.54 (7)	C13—C14—H14C	109.5
C13—O4—Cu1	118.57 (12)	H14A—C14—H14C	109.5
C13—O4—Cu4	120.57 (12)	H14B—C14—H14C	109.5
Cu1—O4—Cu4	92.58 (6)	C13—C15—H15A	109.5
C13—O4—Cu2^i^	126.23 (12)	C13—C15—H15B	109.5
Cu1—O4—Cu2^i^	95.43 (6)	H15A—C15—H15B	109.5
Cu4—O4—Cu2^i^	95.84 (6)	C13—C15—H15C	109.5
O1—C1—C3	110.11 (18)	H15A—C15—H15C	109.5
O1—C1—C2	106.79 (17)	H15B—C15—H15C	109.5
C3—C1—C2	111.1 (2)	C13—C16—H16A	109.5
O1—C1—C4	107.63 (17)	C13—C16—H16B	109.5
C3—C1—C4	110.42 (19)	H16A—C16—H16B	109.5
C2—C1—C4	110.7 (2)	C13—C16—H16C	109.5
C1—C2—H2A	109.5	H16A—C16—H16C	109.5
C1—C2—H2B	109.5	H16B—C16—H16C	109.5
H2A—C2—H2B	109.5		
			
Cu4—Cu1—O1—C1	−125.98 (13)	F3A—C6A—C5—C7	−74.5 (14)
Cu2—Cu1—O1—C1	−128.47 (17)	F2A—C6A—C5—C7	41.9 (15)
Cu1^i^—Cu1—O1—C1	179.20 (14)	O2—C5—C7—F5	168.0 (2)
Cu4^i^—Cu1—O1—C1	137.28 (16)	C8—C5—C7—F5	−72.7 (3)
Cu4—Cu1—O1—Cu2	2.49 (10)	C6—C5—C7—F5	46.9 (4)
Cu1^i^—Cu1—O1—Cu2	−52.33 (5)	C6A—C5—C7—F5	42.5 (9)
Cu4^i^—Cu1—O1—Cu2	−94.24 (6)	O2—C5—C7—F4	46.1 (3)
Cu4—Cu1—O1—Cu4^i^	96.74 (6)	C8—C5—C7—F4	165.4 (2)
Cu2—Cu1—O1—Cu4^i^	94.24 (6)	C6—C5—C7—F4	−74.9 (4)
Cu1^i^—Cu1—O1—Cu4^i^	41.91 (3)	C6A—C5—C7—F4	−79.4 (9)
O4^i^—Cu2—O1—C1	−148.00 (15)	O2—C5—C7—F6	−72.5 (2)
Cu3—Cu2—O1—C1	116.72 (13)	C8—C5—C7—F6	46.8 (3)
Cu1—Cu2—O1—C1	128.12 (17)	C6—C5—C7—F6	166.4 (3)
O4^i^—Cu2—O1—Cu1	83.88 (6)	C6A—C5—C7—F6	162.0 (9)
Cu3—Cu2—O1—Cu1	−11.39 (9)	O2—C5—C8—F8	41.7 (3)
O4^i^—Cu2—O1—Cu4^i^	−3.86 (5)	C7—C5—C8—F8	−77.8 (3)
Cu3—Cu2—O1—Cu4^i^	−99.13 (5)	C6—C5—C8—F8	165.9 (4)
Cu1—Cu2—O1—Cu4^i^	−87.74 (6)	C6A—C5—C8—F8	155.1 (10)
Cu2—Cu3—O2—C5	142.06 (19)	O2—C5—C8—F7	163.5 (2)
Cu4—Cu3—O2—C5	144.91 (14)	C7—C5—C8—F7	44.0 (3)
Cu4—Cu3—O2—Cu2	2.85 (9)	C6—C5—C8—F7	−72.3 (4)
Cu2—Cu3—O3—C9	−143.51 (14)	C6A—C5—C8—F7	−83.1 (10)
Cu4—Cu3—O3—C9	−141.18 (19)	O2—C5—C8—F9	−76.8 (3)
Cu2—Cu3—O3—Cu4	−2.33 (10)	C7—C5—C8—F9	163.7 (2)
Cu4—Cu1—O4—C13	127.18 (16)	C6—C5—C8—F9	47.4 (4)
Cu2—Cu1—O4—C13	128.10 (12)	C6A—C5—C8—F9	36.6 (10)
Cu1^i^—Cu1—O4—C13	−178.62 (14)	Cu3—O3—C9—C11	−176.25 (15)
Cu4^i^—Cu1—O4—C13	−138.56 (12)	Cu4—O3—C9—C11	54.9 (2)
Cu2—Cu1—O4—Cu4	0.92 (9)	Cu3—O3—C9—C12	62.5 (2)
Cu1^i^—Cu1—O4—Cu4	54.20 (5)	Cu4—O3—C9—C12	−66.3 (2)
Cu4^i^—Cu1—O4—Cu4	94.26 (6)	Cu3—O3—C9—C10	−57.8 (2)
Cu4—Cu1—O4—Cu2^i^	−96.12 (6)	Cu4—O3—C9—C10	173.43 (17)
Cu2—Cu1—O4—Cu2^i^	−95.20 (6)	O3—C9—C10—F11	−38.9 (3)
Cu1^i^—Cu1—O4—Cu2^i^	−41.92 (4)	C11—C9—C10—F11	80.2 (3)
Cu4^i^—Cu1—O4—Cu2^i^	−1.86 (7)	C12—C9—C10—F11	−160.7 (2)
Cu1—O1—C1—C3	61.2 (2)	O3—C9—C10—F12	−164.0 (2)
Cu2—O1—C1—C3	−54.5 (2)	C11—C9—C10—F12	−44.9 (3)
Cu4^i^—O1—C1—C3	176.65 (14)	C12—C9—C10—F12	74.2 (3)
Cu1—O1—C1—C2	−178.04 (16)	O3—C9—C10—F10	78.5 (3)
Cu2—O1—C1—C2	66.3 (2)	C11—C9—C10—F10	−162.4 (2)
Cu4^i^—O1—C1—C2	−62.6 (2)	C12—C9—C10—F10	−43.3 (3)
Cu1—O1—C1—C4	−59.2 (2)	O3—C9—C11—F15	−44.5 (3)
Cu2—O1—C1—C4	−174.88 (14)	C12—C9—C11—F15	77.9 (3)
Cu4^i^—O1—C1—C4	56.2 (2)	C10—C9—C11—F15	−163.5 (2)
Cu3—O2—C5—C8	50.8 (2)	O3—C9—C11—F14	−167.8 (2)
Cu2—O2—C5—C8	179.87 (16)	C12—C9—C11—F14	−45.5 (3)
Cu3—O2—C5—C7	169.91 (15)	C10—C9—C11—F14	73.2 (3)
Cu2—O2—C5—C7	−61.0 (2)	O3—C9—C11—F13	73.6 (2)
Cu3—O2—C5—C6	−72.9 (5)	C12—C9—C11—F13	−164.1 (2)
Cu2—O2—C5—C6	56.3 (5)	C10—C9—C11—F13	−45.4 (3)
Cu3—O2—C5—C6A	−57.9 (10)	O3—C9—C12—F17	−39.8 (3)
Cu2—O2—C5—C6A	71.2 (10)	C11—C9—C12—F17	−161.0 (2)
F1—C6—C5—O2	42.4 (6)	C10—C9—C12—F17	80.7 (3)
F3—C6—C5—O2	165.1 (4)	O3—C9—C12—F18	−164.2 (2)
F2—C6—C5—O2	−75.6 (5)	C11—C9—C12—F18	74.7 (3)
F1—C6—C5—C8	−80.1 (5)	C10—C9—C12—F18	−43.7 (3)
F3—C6—C5—C8	42.6 (5)	O3—C9—C12—F16	79.1 (3)
F2—C6—C5—C8	161.9 (4)	C11—C9—C12—F16	−42.0 (3)
F1—C6—C5—C7	161.7 (4)	C10—C9—C12—F16	−160.4 (2)
F3—C6—C5—C7	−75.5 (5)	Cu1—O4—C13—C14	−178.10 (16)
F2—C6—C5—C7	43.7 (4)	Cu4—O4—C13—C14	−65.7 (2)
F1A—C6A—C5—O2	35.3 (16)	Cu2^i^—O4—C13—C14	59.7 (2)
F3A—C6A—C5—O2	159.3 (10)	Cu1—O4—C13—C16	−57.5 (2)
F2A—C6A—C5—O2	−84.4 (13)	Cu4—O4—C13—C16	54.9 (2)
F1A—C6A—C5—C8	−79.1 (14)	Cu2^i^—O4—C13—C16	−179.71 (14)
F3A—C6A—C5—C8	44.9 (13)	Cu1—O4—C13—C15	62.1 (2)
F2A—C6A—C5—C8	161.2 (11)	Cu4—O4—C13—C15	174.48 (14)
F1A—C6A—C5—C7	161.5 (11)	Cu2^i^—O4—C13—C15	−60.1 (2)

Symmetry code: (i) −*x*+1, −*y*+1, −*z*+1.

### Hydrogen-bond geometry (Å, º)

**Table:**

*D*—H···*A*	*D*—H	H···*A*	*D*···*A*	*D*—H···*A*
C3—H3*A*···F2	0.98	2.51	3.457 (7)	164
C3—H3*A*···F2*A*	0.98	2.56	3.539 (18)	173
C14—H14*A*···F15	0.98	2.63	3.571 (3)	162
C14—H14*B*···F4^i^	0.98	2.58	3.554 (4)	173
C15—H15*A*···O2^i^	0.98	2.58	3.438 (3)	146
C15—H15*C*···F3*A*^ii^	0.98	2.64	3.581 (18)	161
C16—H16*A*···F16	0.98	2.57	3.536 (3)	170

Symmetry codes: (i) −*x*+1, −*y*+1, −*z*+1; (ii) −*x*+1/2, *y*+1/2, −*z*+1/2.

## References

[bb1] Anson, C. E., Langer, R., Ponikiewski, L. & Rothenberger, A. (2005). *Inorg. Chim. Acta*, **358**, 3967–3973.

[bb22] Bellow, J. A., Yousif, M., Fang, D., Kratz, E. G. G., Cisneros, G. A. & Groysman, S. (2015). *Inorg. Chem.* **54**, 5624–5633. 10.1021/acs.inorgchem.5b00795 26043187

[bb2] Borup, B., Streib, W. E. & Caulton, K. G. (1997). *Inorg. Chem.* **36**, 5058–5063.

[bb3] Bradley, D. C. (1989). *Chem. Rev.* **89**, 1317–1322.

[bb4] Bruker (2016). *APEX3*, *SAINT* and *SADABS*. Bruker AXS Inc., Madison, Wisconsin, USA.

[bb5] Cripps, W. S. & Willis, C. J. (1975*a*). *Can. J. Chem.* **53**, 817–825.

[bb6] Cripps, W. S. & Willis, C. J. (1975*b*). *Can. J. Chem.* **53**, 809–816.

[bb7] Greiser, T. & Weiss, E. (1976). *Chem. Ber.* **109**, 3142–3146.

[bb8] Håkansson, M., Lopes, C. & Jagner, S. (2000). *Inorg. Chim. Acta*, **304**, 178–183.

[bb9] Krossing, I. (2012). Private Communication (refcode GEQCUC). CCDC, Cambridge, England.

[bb10] Lieberman, C. M., Vreshch, V. D., Filatov, A. S. & Dikarev, E. V. (2015). *Inorg. Chim. Acta*, **424**, 156–161.

[bb21] Lopes, C., Håkansson, M. & Jagner, S. (1997). *Inorg. Chem.* **36**, 3232–3236. 10.1021/ic961287x 11669985

[bb11] McGeary, M. J., Wedlich, R. C., Coan, P. S., Folting, K. & Caulton, K. G. (1992). *Polyhedron*, **11**, 2459–2473.

[bb12] Nikitinsky, A. V., Bochkarev, L. N. & Khorshev, S. Y. (2000). *Russ. Chem. Bull.* pp. 1273–1281.

[bb13] Ohashi, M., Adachi, T., Ishida, N., Kikushima, K. & Ogoshi, S. (2017). *Angew. Chem. Int. Ed.* **56**, 11911–11915. 10.1002/anie.201703923 28585741

[bb14] Purdy, A. P. & George, C. F. (1991). *Inorg. Chem.* **30**, 1969–1970.

[bb15] Purdy, A. P. & George, C. F. (1995). *Polyhedron*, **14**, 761–769.

[bb16] Purdy, A. P. & George, C. F. (1998). *Polyhedron*, **17**, 4041–4048.

[bb17] Sheldrick, G. M. (2008). *Acta Cryst.* A**64**, 112–122. 10.1107/S0108767307043930 18156677

[bb18] Sheldrick, G. M. (2015*a*). *Acta Cryst.* A**71**, 3–8.

[bb19] Sheldrick, G. M. (2015*b*). *Acta Cryst.* C**71**, 3–8.

[bb20] Terry, K. W., Lugmair, C. G., Gantzel, P. K. & Tilley, T. D. (1996). *Chem. Mater.* **8**, 274–280.

